# Validity, Reliability, and Sensitivity of a 3D Vision Sensor-based Upper Extremity Reachable Workspace Evaluation in Neuromuscular Diseases

**DOI:** 10.1371/currents.md.f63ae7dde63caa718fa0770217c5a0e6

**Published:** 2013-12-12

**Authors:** Jay J. Han, Gregorij Kurillo, R. Ted Abresch, Alina Nicorici, Ruzena Bajcsy

**Affiliations:** Department of Physical Medicine and Rehabilitation, University of California, Davis, Sacramento, California, USA; Department of Electrical Engineering and Computer Sciences, University of California at Berkeley, Berkeley, California, USA; Department of Physical Medicine and Rehabilitation, University of California, Davis, Sacramento, California, USA; Department of Physical Medicine and Rehabilitation, University of California, Davis, Sacramento, California, USA; Department of Electrical Engineering and Computer Sciences, University of California at Berkeley, Berkeley, California, USA

## Abstract

Introduction: One of the major challenges in the neuromuscular field has been lack of upper extremity outcome measures that can be useful for clinical therapeutic efficacy studies. Using vision-based sensor system and customized software, 3-dimensional (3D) upper extremity motion analysis can reconstruct a reachable workspace as a valid, reliable and sensitive outcome measure in various neuromuscular conditions where proximal upper extremity range of motion and function is impaired. Methods: Using a stereo-camera sensor system, 3D reachable workspace envelope surface area normalized to an individual’s arm length (relative surface area: RSA) to allow comparison between subjects was determined for 20 healthy controls and 9 individuals with varying degrees of upper extremity dysfunction due to neuromuscular conditions. All study subjects were classified based on Brooke upper extremity function scale. Right and left upper extremity reachable workspaces were determined based on three repeated measures. The RSAs for each frontal hemi-sphere quadrant and total reachable workspaces were determined with and without loading condition (500 gram wrist weight). Data were analyzed for assessment of the developed system and validity, reliability, and sensitivity to change of the reachable workspace outcome. Results: The mean total RSAs of the reachable workspace for the healthy controls and individuals with NMD were significantly different (0.586 ± 0.085 and 0.299 ± 0.198 respectively; p<0.001). All quadrant RSAs were reduced for individuals with NMDs compared to the healthy controls and these reductions correlated with reduced upper limb function as measured by Brooke grade. The upper quadrants of reachable workspace (above the shoulder level) demonstrated greatest reductions in RSA among subjects with progressive severity in upper extremity impairment. Evaluation of the developed outcomes system with the Bland-Altman method demonstrated narrow 95% limits of agreement (LOA) around zero indicating high reliability. In addition, the intraclass correlation coefficient (ICC) was 0.97. Comparison of the reachable workspace with and without loading condition (wrist weight) showed significantly greater RSA reduction in the NMD group than the control group (p<0.012), with most of the workspace reduction occurring in the ipsilateral upper quadrant relative to the tested arm (p<0.001). Reduction in reachable workspace due to wrist weight was most notable in those subjects with NMD with marginal strength reserve and moderate degree of impairment (Brooke = 2) rather than individuals with mild upper extremity impairment (Brooke = 1) or individuals who were more severely impaired (Brooke =3). Discussion: The developed reachable workspace evaluation method using scalable 3D vision technology appears promising as an outcome measure system for clinical studies. A rationally-designed combination of upper extremity outcome measures including a region-specific global upper extremity outcome measure, such as the reachable workspace, complemented by targeted disease- or function-specific endpoints, may be optimal for future clinical efficacy trials.

## Introduction

Most efficacy clinical trials in neuromuscular diseases (NMDs) to date have focused on ambulatory outcome measures as the primary endpoints. However, focusing solely on the ambulatory outcome measures for clinical trials excludes a large portion of potential study populations (e.g. those who are non-ambulatory, older, or at various stages of disease progression not satisfying the mobility criteria). This becomes doubly critical in neuromuscular diseases where majority are also rare conditions with small pool of potential study participants. Thus, one of the major challenges in NMD research has been lack of upper extremity clinical endpoints that can be useful for clinical trials.

Several recent international workshops and studies have highlighted the need to identify and/or develop clinical outcome measures that can be used for efficacy studies in both ambulatory and non-ambulatory NMD populations.^1^^,^ 2 ^,^ 3 ^,^ 4 To make these measures clinically meaningful and suitable for clinical trials, it was recommended that the developed measures should aim to assess a spectrum of upper limb function in non-ambulant patients, as well as being sensitive to changes that may occur in stronger ambulant patients.^5^ Additionally, the developed measures should be easy to obtain in multi-site settings, and be valid, sensitive, and reproducible. Quantitative observer-rated measures that can be standardized are preferred, as long as they have been shown to reflect functional aspects of upper extremities and correlate with activities of daily living (ADLs; such as ability to feed, dress oneself, manage bowel/bladder care, retrieve an object from a countertop or cupboard, use a telephone, remote, or computer, open a door or push an elevator button, dry and comb hair, or wash face).

Many traditional clinical evaluations of upper extremity function are available and include range of motion (ROM), manual and quantitative muscle strength test (MMT, QMT), standardized timed function tests (9-hole peg test, Jebsen-Taylor hand function test) and various motor function scales (Brooke).^6^^,^ 7 ^,^ 8 ^,^ 9 ^,^ 10 ^,^ 11 However, development of a region-specific and global upper extremity outcome metric that can intuitively encapsulate an overall upper extremity functional status would be useful.^12^

A wide range of daily activities require unrestricted movement of the upper extremity, primarily in the shoulder, to extend the reachability of the hand which is used to grasp, position or otherwise interact with various objects and environment.^13^ Therefore, the concept of reachable workspace can be thought to closely associate with an overall functional status of an upper extremity. An upper extremity functional performance measure such as a 3-dimensional (3D) reachable workspace, unlike traditional measurements of isolated muscle strength or joint range of motion, promises to be more sensitive to clinically meaningful changes in an individual’s function as well as activities of daily living.

An in-depth characterization of the proximal upper extremity motion can be obtained using traditional motion analysis systems with active or passive markers (multiple cameras arrayed to provide 360° motion information).^14^^,^ 15 Although quantification and visualization of 3D reachable/functional workspace is achievable through such a motion capture system, large costs and space requirements often relegate their use in laboratory settings rather than clinical trials. Therefore, development of a simple, portable and cost-effective reachable workspace assessment of upper extremity was sought that can be used practically in various NMD clinical and research settings.

Recently, an innovative method to measure and graphically reconstruct an individual’s upper extremity 3D reachable workspace using a low-cost single stereo camera sensor system was developed by the study investigators.^16^ The results showed that a single stereo camera system was capable of capturing and reconstructing 3D reachable workspace that was robust, fast, with minimal loss of data points when compared against full-scale motion analysis system simultaneously. Initial studies also showed that the system was sensitive enough to distinguish between healthy subjects and subjects with varying degrees of upper limb dysfunction due to various NMDs. Since then the methodology to reconstruct and measure 3D reachable workspace has been extended to use with a commercially available and low-cost depth-ranging sensor platform, the Microsoft Kinect.^17^ Other investigators have also begun to examine different clinical applications with the Kinect sensor.^18^^,^ 19 ^,^ 20 ^,^ 21 ^,^ 22

In this paper we assess the applicability of the developed 3D vision sensor-based upper extremity reachable workspace outcome measure by examining the test-retest reliability, validity, and sensitivity to change in neuromuscular disease patients. For validity testing, we assessed the outcome measure’s ability to differentiate individuals with and without functional impairments, and whether it can discriminate between individuals with mild to moderate impairments. To assess the reliability of the system, three repeated trials were performed on the same day for the dominant and the non-dominant arm. To initially assess the sensitivity of the system, we examined whether additional load to the upper extremity (500 gram wrist weight) can alter the total and quadrant 3D reachable workspace envelope surface area. Our hypothesis was that the relatively low weight would not affect the reachability in healthy controls while the patients with shoulder girdle weakness (individuals with neuromuscular conditions with marginal reserve function) would exhibit changes in ability to reach the utmost portions of the workspace envelope (observe most changes in the top quadrants) under the loading condition with a wrist weight. We also examined whether there were gender and hand-dominance differences in upper extremity 3D reachable workspace detectable by the developed stereo-camera sensor system.

## Methods

***Participants.*** A total of 20 healthy individuals (12 female, 8 male; average age: 36.6 ± 13.6 years) and 9 patients (8 male, 1 female; average age: 46.2 ± 16.3 years) with various neuromuscular conditions (5 with Becker muscular dystrophy [BMD], 1 with Duchenne muscular dystrophy [DMD], 1 with Pompe disease, and 2 with facioscapulohumeral muscular dystrophy [FSHD]) were recruited for this study in a regional neuromuscular disease clinic. The study protocol was approved by the University Institutional Review Board for human protection and privacy. Healthy control participants were a convenience sample recruited through IRB approved advertisements and postings. Exclusion criteria for controls were anyone with impairment in arm function that did not allow them to abduct their arms in a full circle until they touch above the head. Anthropometric measurements of arm length for each subject were obtained by determining the distance between the acromion process and the olecranon process and the distance from the olecranon process to the tip of middle finger. This methodology was used to provide the actual limb length and obviate potential measurement errors that can be due to contractures.

Assessment of upper limb functional status was performed with all subjects using the Brooke upper extremity function scale. All control subjects were healthy and had a Brooke grade of 1. Brooke grades and demographic information (diagnosis, age, and sex) for the subjects with upper extremity impairments are shown in Table 1. For reference, grading of Brooke scale with respective functional descriptions is shown in Table 2.

**Table 1. Diagnoses and Brooke grade of the subjects with upper extremity impairments.** 

Id	Age	Sex	Diagnosis	Brooke Grade
1	47	M	BMD	1
2	49	M	BMD	1
3	55	M	BMD	2
4	29	M	BMD	2
5	70	F	FSHD	2
6	29	M	Pompe	2
7	13	M	DMD	2
8	54	M	BMD	3
9	49	M	FSHD	3

**Table 2. Grading for Brooke scale for upper extremities.** 

Grade	Functional description
1	Starting with arms at the sides, the patient can abduct the arms in a full circle until they touch above the head.
2	Can raise arms above head only by flexing the elbow (shortening the circumference of the movement) or using accessory muscles.
3	Cannot raise hands above head, but can raise an 8-oz glass of water to the mouth.
4	Can raise hands to the mouth, but cannot raise an 8-oz glass of water to the mouth.
5	Cannot raise hands to the mouth, but can use hands to hold a pen or pick up pennies from the table.
6	Cannot raise hands to the mouth and has no useful function of hands.

***Experimental Procedures.*** The experimental protocol for sensor system setup and arm movement detection followed the already published protocol. Briefly, subjects were seated in front of a single stereo-camera and underwent standardized upper extremity movement protocol under the supervision of a study clinical evaluator. The standardized simple set of movements consisted of lifting the arm from the resting position to above the head while keeping the elbow extended, performing the same movement in vertical planes at around 0, 45, 90, 135 degrees (i.e. shoulder abduction in coronal plane with origin at ipsilateral shoulder serves as 0 degree, and shoulder forward flexion in sagittal plane would be designated as 90 degree). The second set of movements consisted of horizontal sweeps at the level of the umbilicus and shoulder. The entire sequence of movements was recorded together. The study protocol movements were simple to perform for the subjects and typically took less than 1 minute for the entire sequence of movements; yet, the shoulder underwent its full active ROM (except for the extreme shoulder extension that is limited by the back of the chair). Each set of movements was repeated three times for left and right arm. Subjects were instructed to reach as far as they could while keeping the elbow straight. If they were unable to reach further, they were to return to the initial position and perform the next movement. After a rest period depending on subject’s report of readiness for next set of movements (lasting 1-3 minutes), 500 gram wrist weights were attached by Velcro strap to the wrists of all the subjects, and the above-described set of movements was repeated. During the measurements, the study clinical evaluator (Kinesiologist, AN) demonstrated the movements in front of the subject to dictate the speed and order of movement segments. If the clinical evaluator observed compensatory movements, such as trunk rotation or leaning, the recording was repeated from the beginning with adequate rest breaks.

***3D Data Collection and Reachable Workspace Envelope Analysis.*** As described previously by the investigators,^16^ a BumbleBee2 camera (Point Grey Inc., Richmond Canada) was used to capture the 3D position of the hand via small LED markers attached to participant’s pre-determined anatomical landmarks. In combination with a developed software algorithm for marker detection and tracking, 3D triangulation of hand position and workspace analysis was performed. The tracked 3D hand trajectory was transformed into body-centric coordinate system defined by the four markers on the body and fitted spherical surface. We applied alpha shapes (with radius π/4) to create a concave 2D polygon that tightly fit the data points in order to identify the boundaries of the surface area and to determine the total surface area into four quadrants corresponding to the coordinate system with origin at the shoulder joint, and defined by the standardized human body planes. The sagittal plane through the shoulder joint defined the ipsilateral and contralateral side of the workspace, while the horizontal plane at the shoulder joint defined the top and bottom parts of the workspace quadrants. The quadrants are enumerated as shown in Figure 1.

**Division of the reachable workspace into four quadrants for the left and right arm.**
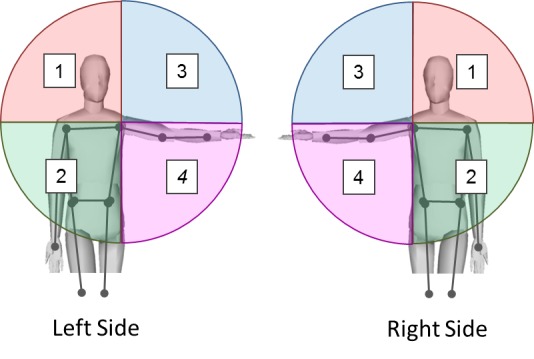

We calculated the absolute reachable workspace surface envelope area (m^2^) for each of the quadrants and the summated total area (m^2^). Normalization of acquired reachable workspace surface areas to the surface area of the unit hemi-sphere allowed comparison between subjects. This relative surface area (RSA) represents the portion of the unit hemi-sphere that is covered by the hand movement. It is determined by dividing the area by the factor \begin{equation*}4\pi r^{2} \times (1/2)\end{equation*}, where *r* represents the arm length. This allows scaling of the data by each person’s arm length, permitting comparison between subjects. The RSA represents the extent of active ROM and reachable workspace of an individual, taking into account the various limitations that can reduce the reachable workspace such as contractures that may be present. For example, with contracture the ‘effective’ radius of the reachable workspace will be smaller and thus will be reflected by correspondingly reduced RSA.

***Statistical Analyses.*** The objectives of this study are to develop and test a new outcome measure that could be used to describe the central tendency and variability of this test in subjects with upper extremity functional impairments as compared to able-bodied controls. This study assesses the validity, reliability, sensitivity, and utility of a newly developed stereo camera-based system to assess upper extremity 3D reachable workspace envelope surface area and relative surface area. We assessed the validity by determining whether subjects with documented impairment in ROM as measured by the Brooke scale (Table 2) would exhibit significantly reduced reachable surface areas than controls, by quadrant and total area. To assess the reliability of the system, three repeated trials were performed on the same day for the dominant and the non-dominant arm. A one-way repeated measures analysis of variance was used to determine the reliability during each activity. Individual error scores of zero indicate reliability. Bland-Altman plots were used to show the dispersion of the individual reliability of the measures. In addition, the coefficient of variation (CV) and the intraclass correlation coefficient (ICC) were calculated further for measurements of reliability. We examined the ICC of each of the three trials and performed Bland-Altmann plots. Initial sensitivity of the system was assessed by examining the response of reachable workspace under non-weighted vs. 500 g loading condition on the wrist in healthy able-bodied individuals and individuals with mild to moderate upper extremity impairments in ROM. An ANOVA of the differences (reachable workspace surface area before the load - workspace surface area after the load) was performed. For all data subgroups, scale scores and variability, descriptive statistics of the task, and test performance were computed. For validity and sensitivity analyses, each of the variables of the three trials was calculated and then averaged for each participant. Data were checked for normality through the Shapiro Wilk’s test. Data meeting the normality assumption (p>0.05) were then analyzed parametrically. Mann-Whitney U test and Kruskal-Wallis ANOVA were used for non-parametric statistical analysis. Differences were determined between subgroups by using an ANOVA or t-tests for two groups. All statistical analyses were conducted in SYSTAT 11.0. For all statistical analyses, a P value of 0.05 was accepted as the level of statistical significance.

## Results

***Validity. ***A typical 3D graphical representation of total reachable workspace as well as quadrant areas of healthy control and subjects with NMDs is shown in Figure 2.

**3D graphical representation of the reachable workspace.**
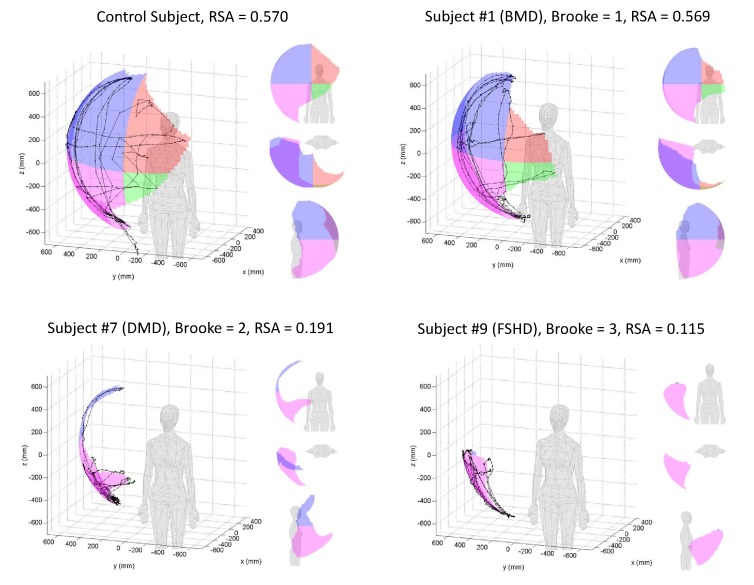
Example graphical outputs of RSA for a healthy individual (control subject), an individual with Becker muscular dystrophy and mild phenotype (BMD, Brooke=1), an individual with Duchenne muscular dystrophy (DMD, Brooke=2), and an individual with Facioscapulohumeral dystrophy (FSHD, Brooke=3).

As shown in Table 3, the total mean relative surface area (RSA) of the subjects with NMD and the healthy controls are significantly different from each other. In addition, the RSA of each quadrant was significantly different between the subjects with NMD and the healthy controls. There was relatively greater loss of upper quadrant reachable workspace (above the shoulder quadrants 1 and 3) in subjects with NMDs as compared to controls, as can be expected.

**Table 3. Mean relative surface area (RSA) of the reachable workspace surface envelope from controls and Subjects with NMD by quadrant and total area.** Means ± SD and sample size (n) are presented as the average value over three repeated trials for dominant and non-dominant sides (there were no significant side to side difference). NMD vs Control by two-sample t-test. Ha: mean = 0

	Quad 1	Quad 2	Quad 3	Quad 4	TOTAL
Subject samples (n)	Contralateral Quadrants Upper & Lower		Ipsilateral Quadrants Upper & Lower		
NMD	0.014 ±0.019 (18)	0.012 ±0.019 (18)	0.122 ±0.107 (18)	0.151 ±0.072 (18)	0.299 ±0.198 (18)
CONTROL	0.070 ±0.031 (40)	0.035 ±0.016 (40)	0.248 ±0.054 (40)	0.234 ±0.031 (40)	0.586 ±0.085 (40)
NMD vs CONTROL	p<0.001	p<0.001	p<0.001	p<0.001	p<0.001

Further analysis was performed to assess the relative surface area (RSA) of the subjects with NMD by documented impairment as determined by the Brooke upper extremity functional grade. An analysis of variance revealed significant differences between the total RSA by Brooke grade of the subjects with NMD (p<0.05). The mean total RSA for subjects with NMD with a Brooke grades of 1, 2, and 3 were 0.493 ± 0.066, 0.294 ± 0.153, and 0.080 ± 0.023, respectively. As shown in Figure 3 with the radar plot, the NMD group with a Brooke grade of 1 had a slightly reduced reachable workspace area than healthy controls with the same Brooke grade of 1, with most appreciable losses in Quad 1 and Quad 3 (upper quadrants). Individuals with Brooke grade of 2 demonstrate reductions in RSA of both the ipsilateral upper and lower quadrants when compared to controls (48% in Quad 3 and 39% in Quad 4 respectively). Individuals who had a Brooke grade of 3 displayed minimal reachable workspace that was essentially limited to the lower ipsilateral quadrant (quadrant 4).

For additional validity testing, two-sample t-tests were used to assess whether gender and hand dominancy affects the total as well as quadrant RSAs; however, these did not have significant effects (data not shown). Briefly, the RSA of Quadrant 2 was statistically greater only for the healthy able-bodied female subjects than males, which may be indicative of their greater flexibility. However, no other significant gender or hand dominancy differences were observed either in the control or NMD groups in the total reachable workspace or in other quadrants.

**Radar plot showing quadrant relative surface area (RSA) by groups.**
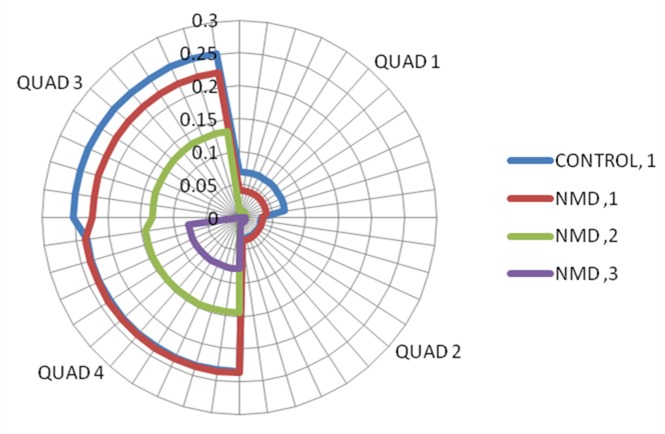
Controls with Brooke grade 1 is shown in blue line, subjects with NMD with Brooke grade 1,2,3 are shown in red, green, and purple lines respectively (shown in right upper extremity orientation).

***Reliability.*** The mean difference in the total reachable surface area for the repeated data collections was small (0.014) and not significantly different from zero (p=0.68) as shown in the Bland-Altman plot in Figure 4. Random errors are scattered in parallel over the measurement range, and there is no evidence for the presence of proportional error. The intraclass correlation coefficient (ICC) for total reachable workspace surface area was 0.97.

**Reliability analysis.**
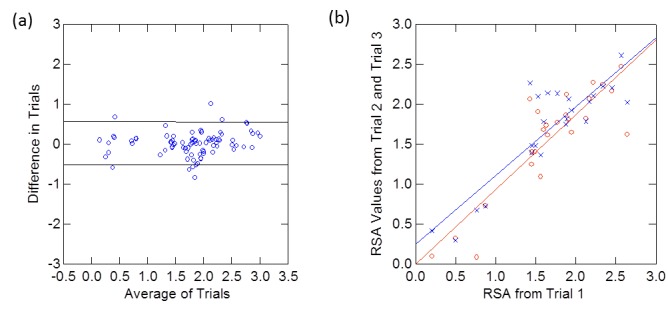
The Bland-Altman plot for the total reachable surface area (a). The mean difference for the groups was 0.014 and the upper lines and lower lines designate the 95% limits of agreement. Reproducibility of the total RSA at trial 2 and trial 3 versus trial 1 (b).

***Sensitivity. ***The sensitivity of the system was assessed by determining whether the methodology would detect a difference in the reachable workspace area with no load and after a small 500 g load was placed on the wrist (Table 4). In the healthy able-bodied subjects, this amount of small wrist weight minimally reduced the RSA in the total reachable workspace, mainly in quadrant 1 (upper contralateral quadrant). In the subjects with NMD even this small load significantly reduced the RSA in quadrants 1 and 3 (upper quadrants) as well as quadrant 4 (ipsilateral lower quadrant). However, there was no statistically significant difference in the total RSA reduction sustained by the control and subjects with NMD. Nevertheless, when the reachable workspace that was lost due to the additional load was expressed as a percentage of the initial pre-load RSA, the NMD group had a significantly greater reduction in percent RSA than the control group (p<0.012). Most of the percent reduction occurred in quadrant 3 (p<0.001). Figure 5 graphically depicts reduction in reachable workspace with the 500 gram load in both controls and subjects with NMDs.

**Table 4 . Reduction in RSA after addition of 500 g load (wrist weight):** Means ± SD. One-sample t-test. p-value represents comparison between RSAs with and without loading condition. ns= not significant. Ha: mean > 0

	Quad 1	Quad 2	Quad 3	Quad 4	TOTAL
	Contralateral Quadrants Upper & Lower		Ipsilateral Quadrants Upper & Lower		
CONTROL	0.011 ±0.026 (39) p<0.007	0.001 ±0.016 (39) ns	0.007 ±0.028 (39) ns	0.007 ±0.026 (39) ns	0.025 ±0.069 (39) p<0.016
NMD	0.004 ±0.007 (17) p<0.016	0.005 ±0.012 (17) ns	0.022 ±0.031 (17) p<0.005	0.018 ± 0.030 (17) p<0.012	0.039 ±0.049 (17) p<0.002

**Radar plot of reduction in reachable workspace (RSA) with loading condition (wrist weight).**
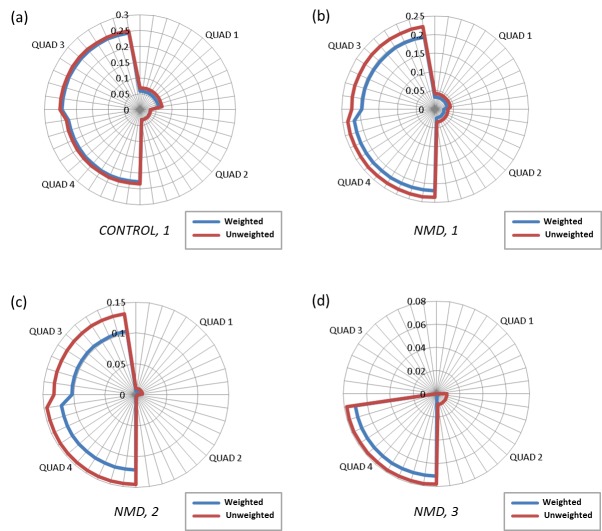
The results of RSA reduction are shown for the following groups: controls (a), NMD subjects with Brooke grade 1 (b), with grade 2 (c), and with grade 3 (d). (Shown in right upper extremity orientation. Note: scale is different for better visualization).

Subjects who had a moderate degree of impairment (Brooke grade = 2) exhibited a greater percent reduction of their original reachable workspace with loading condition than individuals with mild upper extremity impairment (Brooke = 1) or individuals who were more severely impaired (Brooke =3). Analysis of which quadrant most significantly contributed to this reduction in overall RSA showed that quadrant 3 was most affected by the loading protocol (p<0.008). Although the sample sizes are small, the results suggest that reachable workspace measurement in combination with loading condition protocol may provide finer granularity in detection of marginal shoulder weakness and upper extremity impairments.

## Discussion

In our previously published paper, we demonstrated the feasibility of using a relatively inexpensive vision-based sensor system to acquire an upper extremity region-specific outcome measure (reachable workspace) capable of discerning changes in the upper extremity function for a clinical trial. In this paper, we follow up on the previously published work^16^ by evaluating the validity, reliability, and sensitivity of the developed methodology and the outcome measurement. The results support discriminant validity of the developed methodology and outcome measure by demonstrating significant difference in the total reachable workspace RSA between healthy able-bodied controls and patients with NMDs. Furthermore, the results show that the developed RSA outcome measure can provide additional granularity through analysis by side (ipsilateral versus contralateral) or by quadrant (upper versus lower). Depending on the target of the therapeutic treatment and purpose of the efficacy trial, any of these endpoints may be useful as outcome variables to assess changes in the upper extremity range of motion.

Even though the sample sizes were relatively small, the data suggests that the developed system and methodology is not only capable of detecting differences in the total reachable workspace surface area, but also suggest that the system might be sensitive enough to differentiate the changes in reachable workspace of each quadrant between individuals with no documented impairment, mild impairment, and moderate impairment as categorized by Brooke scale. These results will need verification with a larger sample per subgroup. The continuous variable nature of the reachable workspace outcome measure as opposed to the ordinal nature of the Brooke scale will likely contribute to the sensitivity and utility of the developed outcome measure. In addition, through validity testing the methodology, the results reveal, as was suspected, that the individuals with NMD and mild phenotype begin to lose ROM in their upper quadrants first (with ipsilateral upper quadrant undergoing somewhat greater reduction in reachable workspace than the contralateral upper quadrant). The Bland-Altman methodology and the intraclass correlation coefficient indicates a high test-retest reliability.

The sensitivity of the developed measurement system was evaluated by addition of a very small load (500 gram wrist weight). The developed loading condition methodology in combination with the reachable workspace outcome measure was able to detect reduction in reachable workspace with loading conditions in both the healthy control and the subjects with NMD. As we hypothesized, even a relatively small additional wrist weight had a greater effect on the subjects with NMD than on the able-bodied control subjects, as observed by greater reduction of RSA in subjects with NMD. As expected, the upper quadrants’ reachable workspace was reduced to a greater degree than the lower quadrants’. Furthermore, the loading protocol had the most profound effect on individuals who had a Brooke grade of 2 (those individuals with marginal weakness who can raise arms above head only by flexing the elbow, by shortening the circumference of the movement or using accessory muscles). There was less degree of reduction in RSA for the subjects with NMD with Brooke grade 1 and 3, most likely due to presence of adequate reserve strength to overcome the small load in case with Brooke grade 1, and the underlying severity of weakness in those with Brooke grade 3 which suggests a floor effect of the method using the selected weight. Although this study has a very small sample size, it shows that the methodology might be capable of detecting small differences in reachable workspace.

Overall, the results suggest that reachable workspace measurement in combination with alternative loading condition protocols with weights gradually increasing from low to higher weights may further improve the sensitivity of the test, and provide additional granularity to detect subtle differences in upper extremity function found in individuals with various neuromuscular conditions. For this present study, an additional load of 500 g wrist weight was chosen as a standard weight for the protocol, but it was also chosen with consideration for clinical meaning because it is similar to the weight of a glass of water and various office-setting objects. The ability to maneuver this weight in space may have a profound effect on activities of daily living such as being able to handle and drink from a cup/mug, feed independently, or perform work-related duties. The results of the study encourage us to further investigate the effects of various load conditions (i.e., using set of lighter weights) on the reachable workspace envelope that can be used to further quantify the upper extremity function, provide finer granularity to the outcome measure, and assess fatigue effects.

Some of the limitations of this study were the small sample sizes and that the study did not examine individuals who had severe impairments in the arm function with Brooke grade >3. The study’s focus is the reachable workspace outcome measure and in fact, the developed method is most appropriate with individuals with mild to moderate proximal upper extremity impairments, with Brooke grade ≤3. In general with neuromuscular conditions (there are also exceptions), individuals with more profound impairment of their upper extremity function have very little movements at the shoulder. For these instances, other tests will need to be developed and may be more appropriate to assess range of motion and function for the distal upper extremity (forearm, wrist, hand, and fingers).

Future studies involving the developed 3D reachable workspace outcome include further correlation with other established upper extremity measures as well as person-reported outcome measures of function and correlation with clinically-meaningful milestone events such as loss of self-feeding ability. Building on the developed foundational methodology and the concept of reachable workspace outcome measure, the investigators are also developing a Kinect sensor-based system^17^, which promises to be more cost-effective and with greater potential for scalability and sustainability. A rationally-designed combination of upper extremity outcome measures including a region-specific global upper extremity outcome measure, such as the reachable workspace, complemented by targeted disease- or function-specific endpoints, may be optimal for future clinical efficacy trials.

## Conclusions

This study has shown that the developed innovative approach to assessing upper extremity function by reachable workspace outcome measure, using a 3D vision-based sensor system is valid, reliable, and sensitive to small changes in upper extremity range of motion. The 3D reachable workspace surface envelope area as a continuous variable is a direct measurement of overall ROM in the upper extremity, and does alleviate some problems associated with other surrogate measures (e.g. ordinal scale, lack of granularity in data, task-oriented outcomes with limited generalizability, time variable as a sole outcome for performance of complicated tasks). In addition, the developed reachable workspace outcome measure provides an intuitive region-specific global metric for upper extremity that can be used across multiple neuromuscular conditions. Continued progress in development of innovative upper extremity outcome measures will facilitate the overall therapeutic discovery process.

## Competing Interests

The authors have declared that no competing interests exist.
